# Advances in Metabolic Profiling of Biological Samples

**DOI:** 10.3390/metabo13040534

**Published:** 2023-04-09

**Authors:** Joana Pinto

**Affiliations:** 1 Associate Laboratory i4HB, Institute for Health and Bioeconomy, Department of Biological Sciences, Laboratory of Toxicology, Faculty of Pharmacy, University of Porto, 4050-313 Porto, Portugal; jipinto@ff.up.pt; 2 UCIBIO–REQUIMTE, Department of Biological Sciences, Laboratory of Toxicology, Faculty of Pharmacy, University of Porto, 4050-313 Porto, Portugal

Metabolomics constitutes a promising approach to clinical diagnostics, but its practical implementation in clinical settings is hindered by the requirement for rapid and efficient analytical methods. This Special Issue provides valuable insights into the development of novel sample preparation protocols and analytical methods for rapid metabolite analysis in biofluids and tissues. Specifically, a range of articles is presented [1,2,3], each addressing different aspects of this challenge. Campanella et al. [2] proposed a fast and straightforward method for the analysis of saliva by attenuated total reflectance Fourier-transformed infrared spectroscopy (ATR–FTIR) and Raman spectroscopy for large-scale preclinical studies. The effects of saliva collection and processing were investigated by vibrational spectroscopy and liquid chromatography (LC). This study proposed a novel method for saliva collection via the deposition of multiple spots onto low-cost polypropylene sheets, revealing reliable and reproducible ATR–FTIR spectra. Bordanaba-Florit et al. [1] developed a rapid (6 min runtime per sample) and sensitive method for the quantification of steroid hormone compounds (androgens, estrogens, progestogens, and corticoids) by liquid chromatography–high-resolution mass spectrometry (LC–HRMS). The performance of this methodology was tested in several rat tissues (adrenal glands, testis, prostate, liver, and brain), human urine, and urinary extracellular vesicles. Riccio et al. [3] developed a rapid (10 min) analytical method for the analysis of volatile organic compounds (VOCs) in the urine headspace using gas chromatography coupled with ion mobility spectrometry (GC–IMS). The method provided high sensitivity, yielded linearity at the ppb levels, and enabled the identification of 23 molecules (e.g., ketones, aldehydes, alcohols, and sulfur compounds) in a cohort of 115 urine samples. VOCs are molecules released as products of metabolic pathways in the human body, and alterations in their levels have been related to cancer [4,5].

Some authors have explored the ability to extract meaningful information from small sample volumes, which is especially important in clinical settings where sample availability can be limited. He et al. [6] developed a sample preparation method for simultaneously extracting non-polar and polar metabolites from limited amounts of mouse muscle tissues (5–50 mg dry weight). Overall, 109 lipids (e.g., oxylipins, fatty acids, and lysophospholipids) and 62 polar targeted metabolites (e.g., amino acids, sugars, and nucleotides) were successfully detected via ultra-performance liquid chromatography–tandem mass spectrometry (UPLC–MS/MS) and capillary electrophoresis–mass spectrometry (CE–MS). The influence of the sample isolation speed on metabolite stability revealed that rapid (<15 min) muscle tissue collection is crucial, particularly for more oxidative muscles. These findings will be critical for metabolomic mechanistic studies of sarcopenia (the age-related loss of muscle mass and function).

In addition to rapid analysis, the comprehensive characterization of biological samples is also essential. To this end, Bekhti et al. [7] propose superior analytical methods for the more holistic characterization of meconium, including the integration of two complementary LC–HRMS platforms. Overall, up to 229 polar and non-polar metabolites were successfully identified in human meconium at a high confidence level, belonging to amino acids, carbohydrates, nucleosides, and nucleotides, among other chemical classes. This methodology was applied to investigate the progressive evolution of the meconium metabolic profile in healthy newborns during the first three days of life. 

Other articles have focused on the detection of specific metabolite classes [8,9,10,11], such as bile acids, sphingoid bases, fatty acids, and advanced glycation end products (AGEs), which have been implicated in many diseases. Zhang et al. [8] developed and validated a high-throughput method for the comprehensive analysis of bile acids in human and rodent fecal samples. Overall, 58 bile acids were quantified by ultra-high-performance liquid chromatography coupled with mass spectrometry (UPLC–MS). This analytical method was applied to identify and quantify BAs in end-stage renal disease patients. The profiling of bile acids is particularly important for gut-microbiome-related health studies since a complex interplay between bile acids and gut microbiota has been reported [12]. Morano et al. [9] compared different pre-analytical procedures, derivatization protocols, and chromatographic conditions for the quantification of sphingolipids in human plasma via liquid chromatography–tandem mass spectrometry (LC–MS/MS). The authors identified several critical steps for obtaining accurate results, such as single-phase extraction followed by an alkaline methanolysis, the choice of appropriate columns in order to efficiently separate complex sphingolipids and sphingoid bases, and the effectiveness of the derivatization procedure for solely non-phosphorylated species. Comprehensive two-dimensional gas chromatography coupled with mass spectrometry (GC × GC − MS) can be a powerful tool to investigate saturated and unsaturated fatty acids in lipidomics studies, as shown by Bhatt et al. [10]. The authors optimized the derivatization and extraction of multiple classes of fatty acid methyl esters (saturated, monounsaturated, and polyunsaturated) in plasma without requiring in-depth MS/MS investigations. This method successfully distinguished boar-tainted and untainted pigs based on their serum fatty acid compositions. In another study, Yan et al. [11] established an untargeted HILIC–MS method for the comprehensive analysis of AGEs in biological samples (plasma, feces, and urine). The authors tested different columns and mobile phases in Maillard model systems. The proposed method revealed good reproducibility and AGE coverage in the presence of other endogenous metabolites from biological matrices. Elevated levels of AGEs have been associated with several pathologies, including cardiovascular disease, diabetes mellitus, cancer, and Alzheimer’s disease [13].

The metabolite annotation process is a pivotal step in metabolomics and frequently represents a bottleneck in the discovery of biologically relevant metabolites (e.g., biomarkers). In this context, Renai et al. [14] undertook a novel investigation of the pertinence of Feature-Based Molecular Networking (FBMN) in combination with two novel, nutritionally relevant mass spectral libraries (~300 reference molecules) to increase the accuracy of metabolite annotation and explore the postprandial kinetics of the metabolites present in biological samples. This approach enabled the annotation of 67 berry-related and human-endogenous metabolites in the urine from individuals taking *Vaccinium* supplements, revealing similar or higher performance when compared with other annotation workflows. In addition, this tool linked several metabolite classes with phase II (early postprandial) and phase I (late postprandial) metabolism. 

Finally, Viegas et al. [15] integrated measurements of glucose 6-phosphate, pentose phosphate pathway, and de novo lipogenesis fluxes in mice to provide a holistic assessment of hepatic glucose and fructose metabolism. The authors showed that a combination of deuterated water and [U-^13^C]hexose sugar can quantify these fluxes in mice under natural feeding conditions through the ^2^H- and ^13^C-based nuclear magnetic resonance (NMR) analysis of liver glycogen and triglyceride. The results demonstrated that glucose 6-phosphate accounted for 40–60% of lipogenic acetyl-CoA and that 10–12% was oxidized by the pentose phosphate pathway. The NADPH produced from flux in the pentose phosphate pathway accounted for a minority (~30%) of the total de novo lipogenesis requirements. This study provides critical information for understanding hepatic sugar metabolism under pathological conditions.

Overall, this Special Issue provides pivotal methodological and technological advancements for the rapid and comprehensive analysis of metabolites in clinical settings. These advances are crucial for the translation of metabolomics into routine clinical practice and have the potential to revolutionize disease diagnosis and personalized medicine. As a Guest Editor, I would like to thank all the authors for their remarkable studies, the peer reviewers, and the *Metabolites* Editorial Office for their support and contributions.
